# Kuwanon A Targeted YWHAB in Hepatocellular Carcinoma Cells to Inhibit the Raf/MEK/ERK Signaling Pathway

**DOI:** 10.3390/cells14191487

**Published:** 2025-09-23

**Authors:** Jingyang Xu, Hongbo Chang, Yongzhao Wang, Yi Du, Liping Zhong, Hongjuan Cui

**Affiliations:** 1 State Key Laboratory of Resource Insects, Medical Research Institute, Southwest University, Chongqing 400715, China; swuxjy2001@email.swu.edu.cn (J.X.); a1103668795@email.swu.edu.cn (H.C.); wangyz0508@126.com (Y.W.); du.01@foxmail.com (Y.D.); 2 Jinfeng Laboratory, Chongqing 401329, China; 3 State Key Laboratory of Targeting Oncology, Guangxi Medical University, Nanning 530021, China

**Keywords:** Kuwanon A, hepatocellular carcinoma, YWHAB, chemosensitivity

## Abstract

Hepatocellular carcinoma (HCC) is one of the most common malignant tumors worldwide, and the lung is one of the most frequent metastatic sites for HCC. In this study, we aimed to identify a mild active substance in *Morus alba* L. that can inhibit the pulmonary metastasis of HCC and reduce the drug resistance of clinical therapies. Further deepen the understanding of the anti-cancer functions of the mulberry active substances. In this study, we have screened and identified a flavonoid compound extracted from the root bark of the *Morus alba* L. named Kuwanon A (KA). Our research demonstrated that KA directly targeted the YWHAB (tyrosine 3-monooxygenase/tryptophan 5-monooxygenase activation protein beta) and mediated its dimer dissociation. Thereby inhibiting the MAPK pathway and affecting downstream biological functions, including cell cycle arrest and migration/invasion inhibition. The experiment results proved that KA could inhibit the proliferation and metastasis of highly metastatic HCC cells both in vitro and in vivo. Additionally, when KA was combined with the clinical drug sorafenib, it exhibited a synergistic effect in inhibiting HCC cell proliferation, migration, and invasion. In conclusion, KA demonstrated a favorable anti-tumor effect in HCC cells.

## 1. Introduction

Hepatocellular carcinoma (HCC) is one of the most prevalent malignant tumors worldwide and an important cause of cancer-related mortality, accounting for 8.3% and ranking as the third leading cause of all cancer-related deaths [1]. It is particularly prevalent in regions with high rates of chronic hepatitis B and C infections, such as Asia and Africa [2,3]. The primary risk factors for HCC include chronic hepatitis B virus (HBV) and hepatitis C virus (HCV) infections, cirrhosis, long-term alcohol consumption, aflatoxin exposure, and non-alcoholic fatty liver disease (NAFLD) [4]. Despite recent advances in diagnostic techniques and treatment options, the overall survival rate for HCC patients remains low, largely due to the high frequency of metastasis and recurrence. The most common sites of HCC metastasis include the lungs, lymph nodes, and bones, with pulmonary metastasis being particularly frequent [5]. Therefore, identifying effective therapeutic strategies to inhibit HCC metastasis and improve patient outcomes is of utmost importance.

The mulberry tree, scientifically named *Morus alba* L., is commonly known as “mulberry” or “sang shu”. Originally native to China, this versatile plant has been widely cultivated and naturalized across many countries. It is highly valued as an important herbal resource [5]. The mulberry tree has a long-standing history in traditional Chinese medicine and is renowned for its extensive health benefits. These benefits include antioxidant, cholesterol-lowering, anti-atherosclerotic, weight management, blood sugar regulation, immune-modulating, lipid-lowering, neuroprotective, and liver-protective effects [6]. These therapeutic properties are primarily due to the diverse bioactive compounds found within the plant, such as flavonoids, anthocyanins, phenolic acids, flavonols, and volatile aromatic compounds [7].

*Morus alba* L., including Kuwanon derivatives such as Kuwanon C and G, has shown anticancer activity [8]. Nevertheless, Kuwanon A (KA) has not been extensively and in-depth investigated for its anticancer activity yet. Previous studies have demonstrated that KA induces cytotoxic endoplasmic reticulum stress and apoptosis in gastric cancer cells and enhances the sensitivity of tumor cells to the clinical drug 5-fluorouracil [9]. Additionally, KA has been proven to inhibit the growth of melanoma cells [10]. These findings suggest that KA has potential as a therapeutic agent for various cancers. However, the specific mechanisms by which KA exerts its anticancer effects, particularly in hepatocellular carcinoma, remain to be fully elucidated.

The *14-3-3* gene encodes a member of the 14-3-3 protein family, which is involved in a wide range of cellular processes, including cell cycle regulation, apoptosis, and signal transduction [11]. YWHAB, whose full name is tyrosine 3-monooxygenase/tryptophan 5-monooxygenase activation protein beta, is a member of the 14-3-3 protein family. It is known to interact with various proteins and modulate their functions through protein-protein interactions [12]. In cancer, YWHAB has been implicated in promoting cell proliferation, survival, and metastasis [13,14]. High expression levels of YWHAB have been associated with poor prognosis in several types of cancer, including hepatocellular carcinoma [15]. By regulating key signaling pathways such as the EGFR/PI3K/AKT pathway, YWHAB can influence tumor progression and resistance to therapy [16,17,18]. Therefore, targeting YWHAB represents a promising strategy for cancer treatment.

In this study, we screened 623 mulberry metabolites and identified Kuwanon A (KA) as an active compound that effectively inhibits the progression of hepatocellular carcinoma (HCC) and suppresses the metastasis of highly metastatic HCC cells both in vitro and in vivo. Our results demonstrate that KA specifically targeted YWHAB and mediated the dissociation of its dimer. Thereby affecting downstream biological functions. The phosphorylation signaling cascade of the classical MAPK pathway, including Raf/MEK/ERK, is significantly inhibited [12,19]. Then it led to cell cycle arrest and the inhibition of migration and invasion. When combined with the clinical drug sorafenib, KA exhibits a synergistic effect in inhibiting HCC cell proliferation, migration, and invasion. These findings collectively suggest that Kuwanon A can act as a MAPK signaling pathway inhibitor and exert antitumor effects in HCC. KA held significant promise as a potential anticancer agent for cancer therapy.

## 2. Materials and Methods

### 2.1. Reagents and Antibodies

Kuwanon A (CAS No. 62949-77-3) was procured from LEMEITIAN MEDICINE (Chengdu, China) and dissolved in DMSO. Sorafenib (CAS No. 284461-73-0) was obtained from MedChemExpress (Monmouth Junction, NJ, USA). The antibodies anti-cyclin D1 (Cat No. 26939-1-AP), anti-cyclin E2 (Cat No. 11935-1-AP), anti-CDK 4 (Cat No. 11026-1-AP), anti-cyclin B1 (Cat No. 55004-1-AP), anti-CDK1 (Cat No. 19532-1-AP), anti-ERK 1/2 (Cat No. 11257-1-AP), anti-Phospho-ERK1/2 (Thr202/Tyr204) (Cat No. 28733-1-AP), anti-c-Myc (Cat No. 10828-1-AP), anti-Alpha Tubulin (Cat No. 80762-1-RR), anti-Lamin A/C (Cat No. 10298-1-AP), anti-MYC tag (Cat No. 60003-2-Ig), and anti-DYKDDDDK tag (Cat No. 20543-1-AP) were sourced from Proteintech (Wuhan, China). Additional antibodies, including anti-MEK 1/2 (Cat#9122S), anti-Phospho-MEK 1/2 (Ser217/221) (Cat#9121S), anti-N-Cadherin (Cat#13116T), and anti-E-Cadherin (Cat#3195T) were acquired from Cell Signaling Technology (Boston, MA, USA). The antibody anti-YWHAB (Cat No. ab32560) was acquired from Abcam (Cambridge, UK). MTT (Cat#M5655) and DMSO (Cat#D5879) reagents were purchased from Sigma–Aldrich (St. Louis, MO, USA). Reagents such as DAPI (Cat#C1002), the Cell Counting Kit-8 (Cat#C0038), the Matrix-Gel™ Basement Membrane Matrix (Cat#C0371-1ml), the BeyoClick™ EdU Cell Proliferation Kit with Alexa Fluor 488 (Cat#C0071S), the BCA Protein Assay Kit (Cat#P0012), the Hematoxylin and Eosin Staining Kit (Cat# C0105M), the Nuclear and Cytoplasmic Protein Extraction Kit (Cat#P0027), the Cell Cycle and Apoptosis Analysis Kit (Cat#C1052), the Cell Lysis Buffer for Western and IP (Cat#P0013), the RIPA Lysis Buffer (Cat#P0013B), the Crystal Violet Staining Solution (Cat#C0121), HRP goat anti-mouse antibody (Cat#A0126), HRP goat anti-rabbit antibody (Cat#A0208), and Alexa Fluor 488-labeled Goat Anti-Rabbit IgG (Cat#A0423) were sourced from Beyotime (Shanghai, China). The Blue/Clear Native PAGE Electrophoresis Kit (Cat#RTD6140) was acquired from Real Times (Beijing, China). The Epoxy-Activated Sepharose 6B (Cat#17048001) was obtained from Cytia (Washington, DC, USA). Finally, the transfection reagent Lipofectamine™ 2000 was purchased from Thermo Fisher Scientific (New York, NY, USA).

### 2.2. Cell Culture

HCC cell lines (MHCC97 H and SMMC 7721), normal human liver cells (L-O2), and human embryonic kidney cells (HEK293T, HEK293FT) were purchased from the American Type Culture Collection (University Blvd Manassas, VA, USA). All cell lines were confirmed to be mycoplasma-free and cultured at 37 °C and 5% carbon dioxide.

### 2.3. Cell Proliferation Analysis

Cell viability was assessed using MTT experiments. Cells, together with KA, were seeded into 96-well plates at a density of 2000 cells per well with three replicates per condition. DMSO was used as a control. At designated time points, 20 μL of MTT (Sigma, St. Louis, MO, USA) solution was added to each well and incubated for 3 h. After removing the culture medium, 200 μL of DMSO was added to dissolve the formazan crystals. The absorbance was then measured using a microplate reader at a wavelength of 560 nm.

### 2.4. Colony Formation Assay

Using a colony formation assay, the effect of KA on the colony-forming ability of HCC cells was evaluated. A total of 1000–2000 cells, along with KA, were seeded into each well of a six-well plate. During this period, fresh culture media and KA were replaced multiple times. After 2 to 3 weeks of incubation, the colonies were stained with crystal violet (Beyotime, Shanghai, China) and subsequently quantified in each well using ImageJ 1.52a software.

### 2.5. CCK8 Assay

The CCK8 assay is used to investigate the effect of compounds on cell viability by measuring the amount of formazan dye. Firstly, 8000 cells were inoculated into each well of the 96-well plate with three replicates per condition. At the same time, add the corresponding mulberry active substances. After 48 h of incubation, 20 μL of the Cell Counting Kit-8 (CCK8) reagent (Beyotime, Shanghai, China) was added to each well. After being cultured under 37 °C and 5% carbon dioxide for 2–3 h, the absorbance was measured at 450 nm.

### 2.6. Transwell Assay

The Transwell assay measured the migration and invasion of cells by calculating the number of cells that passed through the material. Prior to the Transwell migration assay, cells were serum-starved in FBS-free culture medium for 24 h. The cells were then detached using trypsin, washed 1–2 times with PBS, and resuspended in DMEM. Approximately 50,000–100,000 cells were seeded into the upper chamber of the Transwell insert. The lower chamber was filled with 600 μL of complete medium containing the test compound. The Transwell system was incubated for 12 h under standard culture conditions (37 °C, 5% CO_2_). After incubation, the migrated cells on the lower membrane surface were fixed with 4% paraformaldehyde (PFA) and stained with 0.1% crystal violet. Non-migrated cells remaining in the upper chamber were carefully removed with a cotton swab. Finally, the stained cells were visualized under a microscope. If matrix gel was needed, it should be added according to the instructions (Beyotime, Shanghai, China) before the cells were seeded.

### 2.7. EdU Staining

EdU staining was employed to assess cell proliferation following the manufacturer’s guidelines (Beyotime, Shanghai, China). A total of 2 × 10^4^ cells, together with KA or DMSO, were seeded into 24-well plates and cultured for 48 h. Subsequently, the cells were incubated with 10 mM EDU for 2 h, followed by fixation with 4% PFA for 15 min, permeabilization with 0.3% Triton X-100 for 10 min, and reaction with Click reaction cocktails for 30 min. Finally, the nuclei were stained with DAPI for 30 min at room temperature before microscopic examination.

### 2.8. Flow Cytometry

Cells were digested, centrifuged, and resuspended in PBS buffer following 48 h of treatment with either KA or DMSO. Prior to cell cycle analysis, the cells were fixed in 75% ethanol for a minimum of 24 h, followed by labeling with PI and RNase (Beyotime, Shanghai, China). Subsequently, the cells were analyzed using flow cytometry. Each group consisted of three replicates.

### 2.9. Transfection and Infection

Short hairpin (sh) RNA targeting YWHAB was cloned into the pLKO.1-puro plasmid, with the sequences listed in Supplementary Table S1. Flag-tagged YWHAB, MYC-tagged YWHAB, and Flag-tagged Raf1 plasmids were purchased from Miaoling (Wuhan, China). Transfection was performed by introducing the specified plasmids into HEK293FT cells using Lipofectamine 2000 (Thermo, New York, NY, USA), following the manufacturer’s instructions. Viral supernatant or cells were collected two days post-transfection. For infection, HCC cells were treated with viral supernatant and polybrene (Sigma, Virginia Beach, VA, USA). After two rounds of infection, the cells were stably selected by using puromycin (Sigma, Virginia Beach, VA, USA).

### 2.10. Western Blot

Cells were harvested using a cell scrape and washed three times with PBS buffer, then lysed on ice in RIPA lysis buffer (Beyotime, Shanghai, China). Protein concentration was determined using a BCA protein assay kit (Beyotime, Shanghai, China). Subsequently, 60 µg of protein was mixed with 5× loading buffer and heated in a 100 °C water bath for 15 min. The samples were subjected to SDS-PAGE electrophoresis, followed by transfer onto a PVDF membrane (Millipore, Darmstadt, Germany). The membrane was blocked with 5% BSA for 2 h at room temperature and incubated sequentially with primary and HRP-conjugated secondary antibodies. Finally, signal detection was performed using an ECL detection system (Clinx, Shanghai, China).

### 2.11. Label Free Analysis

After two days of treatment with KA or DMSO, MHCC97 H cells were collected and analyzed using label-free quantitative proteomic analysis conducted by Shanghai Applied Protein Technology Biotechnology Corporation. The process involved protein extraction, peptide enzymatic hydrolysis, liquid chromatography-tandem mass spectrometry (LC-MS/MS) data collection, and database retrieval. To identify proteins with significant differences between groups, the criteria for screening included a fold change (FC) greater than 2.0 (upregulated > 2.0 or down-regulated < 0.5) and a *p*-value of less than 0.05.

### 2.12. Molecular Docking and PyMOL Visualization

The 3D structure of the protein was downloaded from the RCSB Protein Data Bank (https://www.rcsb.org/) (accessed on 18 May 2024), and the molecular structure of KA was obtained from TCMSP (https://www.tcmsp-e.com/) (accessed on 18 May 2024). In PyMOL, we removed the ligands from the protein and then used them for drug pocket prediction on the website (https://playmolecule.org/deepsite/) (accessed on 18 May 2024). According to the potential drug pocket, the molecular docking was performed in Autodock. We selected the docking results with lower binding energy and more hydrogen bonds. After exporting the structure of the docking result, we conducted the visual analysis in PyMOL.

### 2.13. Animal Studies and Animal Ethics

Thirty-six female BALB/c-nu mice, four weeks old (supplied by Slike Jingda Laboratory Animal Co., Ltd., Changsha, China; Animal qualification number: SCXK-2019-0004), were housed in an SPF room. Based on previous experimental observations, female mice exhibited less individual variability and lower aggression, particularly after tumor inoculation. Moreover, they displayed stable immune responses and lower rejection reactions. Therefore, female mice were selected for this study; 12 mice were used to establish the hepatocellular carcinoma xenografts. MHCC97 H cells (1 × 10^5^ cells) were slowly injected into the left lobe of the mouse liver after being anesthetized with isoflurane. Three days later, the mice received intraperitoneal injections of KA (30 mg/kg) every two days for 45 days. Control mice were administered DMSO injections. Recorded the survival status of the animals. Prior to liver collection, the mice were anesthetized with isoflurane to minimize pain and euthanized by cervical dislocation. Ethics approval serial number: IACUC-20240613-03. Animal experiments were conducted in accordance with the Guide for the Care and Use of Laboratory Animals (Ministry of Science and Technology of China, 2006).

### 2.14. Hematoxylin-Eosin Staining

The liver tissue was fixed in 4% PFA and then prepared into paraffin sections through tissue dehydration, transparency, and wax dip. After baking the slices in the oven, they were dewaxed in xylene and then rehydrated with a series of ethanol gradients. After staining the nuclei with hematoxylin, the slices were differentiated with HCL for a few seconds. After being blued in ammonia solution, the eosin stain was used to stain the cytoplasm of the tissue slices. Lastly, the slices were dehydrated and covered.

### 2.15. Immunohistochemistry

We prepared the tissue slices until rehydration as described in 2.14. Subsequently, we restored the antigen conformation and blocked endogenous peroxidase. The tissue slices were incubated with the corresponding primary antibody overnight after being blocked with 5% goat serum. The diaminobenzidine (DAB) reaction was conducted after incubation of the secondary antibody at room temperature. Subsequently, the nuclei of tissue slices were stained with hematoxylin. After the dehydration, the tissue slices were covered and observed.

### 2.16. Cell Wound Healing Assay

We seeded the cells in 6-well plates and allowed them to adhere and grow to full confluence. Then, we gently scratched the cell monolayer in a straight line using a sterile pipette tip. We rinsed the wells with PBS to remove detached cells and debris. We captured bright-field images of the scratched area at baseline (0 h). We replaced the medium with DMEM containing KA or DMSO and continued incubation. We re-imaged the same wound areas at 24 h and 48 h post-scratch. We quantified cell migration by measuring wound closure (%) over time using image analysis software.

### 2.17. Cell Thermal Shift Assay (CETSA)

The cells were treated with a high concentration (3–4 times the IC_50_) of the drug for 2–4 h before being digested with trypsin. Then the cells were washed 1–2 times with PBS. Resuspend the cells in 450 µL of PBS. Subsequently, divided them into eight portions, each containing 50 µL. Set the appropriate temperature range and gradient. In our research, the range of 44–65 °C was used. Each portion of cells was treated at the specified temperature for 3–5 min. Then, the cells were repeatedly frozen and thawed using liquid nitrogen. After high-speed centrifugation (20,000× *g*, 20 min), we took the top layer of supernatant. After adding an appropriate amount of loading buffer, we conducted the Western Blot assay. Quantitative analysis was used to determine whether the compound has enhanced the thermal stability of the target protein.

### 2.18. Native-PAGE

Blue/Clear Native PAGE (Real Times, Beijing, China) is an electrophoretic technique used to separate protein complexes from biological samples. Instead of SDS, Coomassie Brilliant Blue G-250 binds to the protein, imparting a negative charge, allowing separation in the PAGE gel based on the molecular weight of the proteins. HCC cells treated with KA or DMSO for 48 h were collected using a cell scraper and lysed on ice in IP cell lysis buffer for one hour. A suitable amount of 4× BN/CN-PAGE protein loading buffer and 5% G-250 stain (for loading) was added to the supernatant of the protein sample, followed by BN-PAGE electrophoresis in the Native PAGE gel. After electrophoresis and membrane transfer, the PVDF membrane (Millipore, Darmstadt, Germany) was blocked with 5% BSA for two hours at room temperature. HPR-linked primary and secondary antibodies were incubated for detection, and protein bands were visualized using the ECL detection system (Clinx, Shanghai, China)

### 2.19. Immunofluorescence

Immunofluorescence was used to explore the effect of KA or Sorafenib on the nuclear translocation of ERK. After laying the glass coverslip in 24-well plates, the cells were seeded. Cells were treated with a certain concentration of KA or Sorafenib for 48 h. Firstly, fix it with 4%PFA, and then permeabilize with 0.2% Triton X-100. After blocking with 5% goat serum, the primary antibody was incubated overnight. The next day, incubate with the fluorescent secondary antibody (Alexa Fluor 488-labeled Goat Anti-Rabbit IgG, Beyotime, Shanghai, China) of the same genus. Confocal observation of fluorescence signals was conducted after staining the nucleus with DAPI.

### 2.20. Nuclear and Cytoplasmic Protein Extraction

After treating HCC cells with KA or DMSO, the cells were collected by cell scraping. Adding an appropriate amount of PMSF and reagent A (Nuclear and Cytoplasmic Protein Extraction Kit, Beyotime, Shanghai, China). Vortexed and then ice bathed for 15 min. Adding the reagent B provided in the kit mentioned before. Then vortexed and ice bathed for 1 min. Vortexed again. Centrifuged at 14,000× *g* for 5 min. The supernatant was cytoplasmic protein. Adding a certain amount of nuclear protein extraction reagent to the precipitate. Multiple vortices within 30 min. Centrifuged at 14,000× *g* for 10 min. The obtained supernatant was the nuclear protein.

### 2.21. In Vivo Imaging

MHCC97 H cells labeled with luciferase (1 × 10^5^ cells) were injected into the 4- to 5-week-old female BALB/c-nu mice (SPF grade, average weight 16–18 g) through intravenous injection. Following the injection, luciferase substrate was delivered to the mice via intraperitoneal injection. After a 5-min period of anesthesia with isoflurane, the mice were imaged using an in vivo small animal imaging system. The drug treatment was the same as previously mentioned (2.13), and DMSO was used as a control. To monitor the progression and location of the HCC cells, in vivo imaging was conducted on days 0, 14, 21, and 35 after cell injection.

### 2.22. Epoxy-Activated Sepharose 6B Affinity Media

Epoxy-activated Sepharose 6B (Cytia, Washington, DC, USA) affinity media was used to bind KA molecules following the media’s absorbency swelling. The Sepharose 6B media was co-incubated with KA or DMSO and gently agitated overnight at room temperature. The product was then thoroughly washed through at least three cycles. Any remaining active groups were blocked using 1 M ethanolamine, incubated for 4 h at 37 °C. The 6B media, now bound with KA or DMSO, were further incubated with the cell lysate sample overnight, with gentle agitation at 4 °C. The media was washed again through a minimum of three cycles. A suitable amount of loading buffer was added, and the samples were heated in a 100 °C water bath for 15 min. The resulting supernatant was then ready for use in SDS-PAGE electrophoresis.

### 2.23. Statistics Analysis

All data were presented independently three times and analyzed using GraphPad Prism 8. Results were expressed as Mean ± SD. Statistical significance was assessed for independent samples using the Student’s unpaired *t*-test, with significance defined as *p* < 0.05 (*p* < 0.05: *, *p* < 0.01: **, *p* < 0.001: ***).

## 3. Results

### 3.1. Screening of Compounds with Antiproliferative and Anti-Migratory Effects on Highly Metastatic HCC Cells

**Figure 1:**
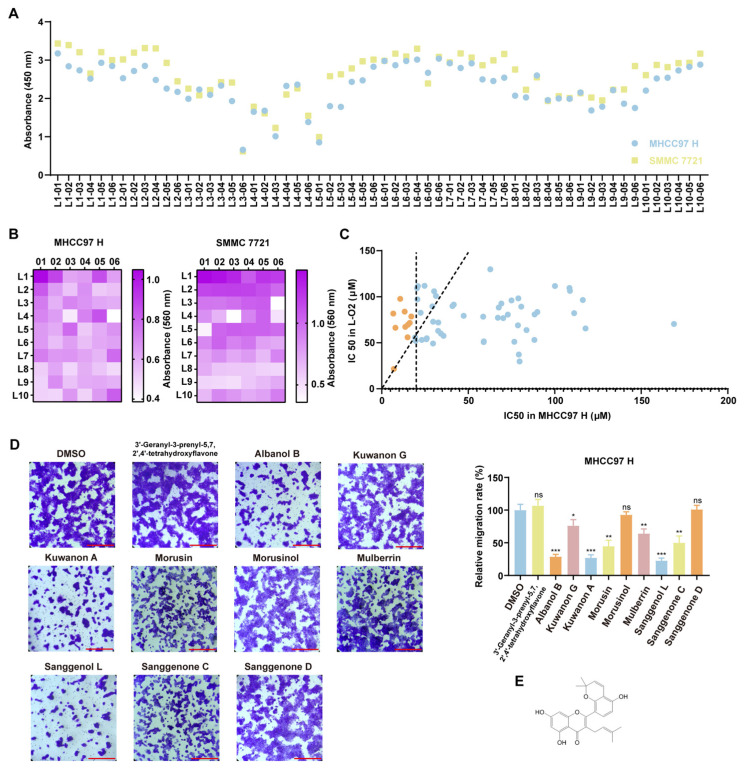
Kuwanon A was found by screening from the mulberry active substance bank. (**A**) The CCK8 assay was used to determine the effects of every drug on cell viability. The indicated concentration (10 µM) of compounds was used to treat MHCC97 H and SMMC 7721 for 48 h. The vertical axis represented the absorbance at 450 nm, and the horizontal axis represented the number of different mulberry active substances. The L1-01 (DMSO) was used as a control. (**B**) To detect the effects of every compound on the colony formation ability of HCC cells (MHCC97 H and SMMC 7721). The indicated concentration (10 µM) of compounds was used to treat HCC cells for 2–3 weeks. Crystal violet solution was used to stain the cell clones. After washing the background with PBS buffer, the color was eluted with absolute ethanol, and the absorption was determined at 560 nm using a microplate reader. Each square represented a different compound. The labels on the horizontal and vertical axes indicated the NO. The L1-01 (DMSO) was used as a control. (**C**) Measured the IC_50_ of each compound in HCC cells (MHCC97 H) and normal liver cells (L-O2) by the MTT method. Each dot (blue and orange dots) represented a different compound. The horizontal axis indicated the IC_50_ value in MHCC97H, and the vertical axis represented that in L-O2. The slant dotted line indicated that the ratio of IC_50_ in normal liver cells compared to IC_50_ in tumor cells was 3. The vertical dotted line indicates an IC_50_ equal to 20 µM in tumor cells. Picked the dots (orange dots) where ratios greater than 3 and the IC_50_ value in tumor cells less than 20 µM, which means the compound not only restrains the proliferation of tumor cells, but also does not harm normal liver tissue. (**D**) Detected the migration inhibition ability of selected compounds by Transwell assay. Further screening of the drug that can suppress the migration of highly metastatic HCC cells (MHCC97 H). The DMSO was used as a control. Scale bars = 200 μm (**E**) The chemical structure of Kuwanon A (Hereinafter referred to as KA). All data are shown as the means ± SD; * *p* < 0.05, ** *p* < 0.01, *** *p* < 0.001. ns: not significant.

Firstly, we found 623 kinds of mulberry active substances from the TCMSP website (https://www.tcmsp-e.com/) (accessed on 26 February 2024). There were 194 kinds in *Mori cortex*, 46 in *Herba taxilli*, 91 in *Mori fructus*, 269 in *Mori follum,* and 23 in *Ramulus mori*. Based on the data of relative molecular weight, AlogP value, Hdon value, Hacc value, and drug-like properties, a total of 59 mulberry active substances were screened out (Supplementary Table S2). To identify the target compound, we tested the effects of 59 traditional Chinese medicine extracts on the proliferation and migration of HCC cells. Using the CCK8 assay, we evaluated the cell viability of highly metastatic HCC cell lines MHCC97H and SMMC 7721 after treatment with 10 μM of the compounds [20,21] (Figure 1A). Results showed that several compounds, such as L3-06 (Sanggenol L), L4-03 (Sanggenone C), L4-06 (Kuwanon A), and L5-01 (Albanol B), significantly inhibited the viability of HCC cells. Subsequently, colony formation assays were used to detect the effects of all compounds (10 μM) on colony formation ability in vitro. The heat map revealed that these compounds also suppressed the colony formation of HCC cells (Figure 1B). Furthermore, we analyzed the half-maximal inhibitory concentrations (IC_50_) of 59 compounds in MHCC97H and L-O2 cells. Then we selected the compounds that had a low IC_50_ value in HCC cells and a weak cytotoxicity at the same time. Finally, we identified 10 compounds with an IC_50_ below 20 µM in MHCC97 H, and the ratio of IC_50_ in normal liver cells compared to tumor cells was greater than 3. These compounds included 3′-geranyl-3-isoprenyl-5,7,2′,4′-tetrahydroxyflavone (L8-06), Albanol B (L5-01), Kuwanon G (L8-04), Kuwanon A (L4-06), Morusin (L9-02), Morusinol (L8-05), Mulberrin (L4-01), Sanggenol L (L3-06), Sanggenone C (L4-03), and Sanggenone D (L4-02) (Figure 1C, Supplementary Figure S1). Transwell assays were used to evaluate the inhibitory effect of these compounds at an IC_50_ concentration on cell migration capacity and further narrowed down the filtering range. The results showed that Albanol B, Kuwanon A, and Sanggenol L significantly inhibited the migration of MHCC97 H (Figure 1D). Because the IC_50_ of Albanol B in normal liver cells was relatively low, and considering its cytotoxicity, Albanol B was not selected. The anti-tumor mechanism of Sanguanol L in HCC was investigated in another study, while this research focused on Kuwanon A and investigated its effects on highly metastatic HCC cells. The chemical structure of KA is illustrated in Figure 1E.

### 3.2. KA Inhibited HCC Cell Proliferation In Vitro and Suppressed Metastasis In Vivo

**Figure 2:**
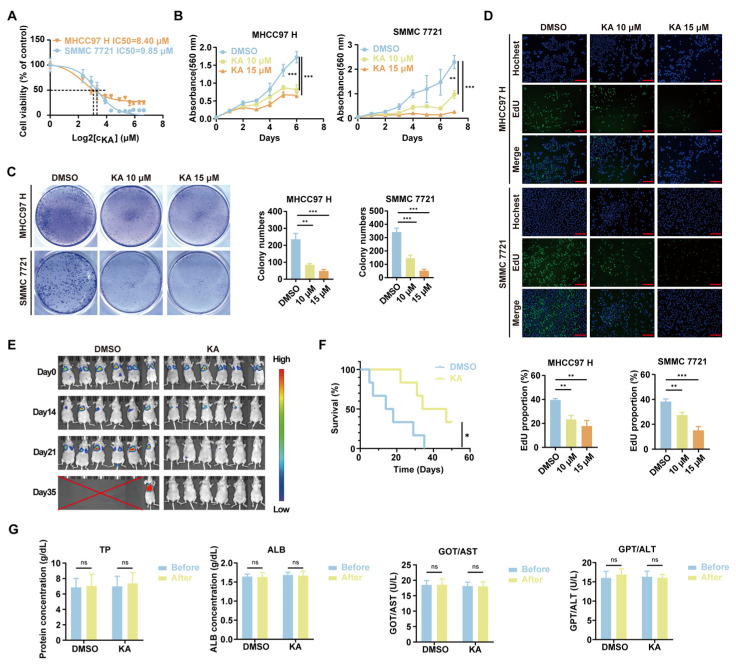
Kuwanon A inhibited the proliferation and metastasis of HCC cells. (**A**) Determine the IC50 of KA in HCC cells (MHCC97 H and SMMC 7721) by the MTT method. The gradient concentration of KA (0, 5, 7.5, 10, 15, 20, 30, 40, 50, 60, 80, 100 μM) treated cells for 48 h, followed by MTT assay to detect the cell viability. The IC50 of KA in MHCC97 H is 8.40 μM, and it is 9.85 μM in SMMC 7721. (**B**) 10 μM and 15 μM KA were used, respectively, to determine their proliferation inhibition ability in HCC cells (MHCC97 H and SMMC 7721). MTT assay was used to detect the cell viability after KA treatment for 1, 2, 3, 4, 5, 6, and 7 days. The DMSO groups were used as controls. (**C**) The influence on colony formation ability in vitro was detected after HCC cells (MHCC97 H and SMMC 7721) were treated with the indicated concentration of KA. The DMSO groups were used as controls. (**D**) The EdU assay was used to determine the amplification ability of HCC cells (MHCC97 H and SMMC 7721) after treatment with the indicated concentration of KA for 48 h. The DMSO groups were used as controls. Scale bars = 200 μm (**E**) The MHCC97 H-Luc cells labeled with luciferase were injected into the BALB/c-nu mice through the tail intravenous injection. Then the mice were imaged every 1–2 weeks using an in vivo small animal imaging system after the intraperitoneal injection of luciferase substrate. During this time, KA or DMSO was delivered by intraperitoneal injection (30 mg/kg/day) every two days. The crossed red line indicated that the mice had died at this moment. (**F**) Displayed the survival curve of the orthotopic xenograft mice. The MHCC97 H were injected into the liver of BALB/c-nu mice. Three days later, KA or DMSO was delivered by intraperitoneal injection (30 mg/kg/day) every two days. The DMSO group was used as a control. (**G**) Compared the changes of ALB and total protein level, as well as the activity of GOT/AST and GPT/ALT, before and after KA injection. The DMSO group was used as a control. All data are shown as the means ± SD; * *p* < 0.05, ** *p* < 0.01, *** *p* < 0.001. ns: not significant.

To determine the antiproliferative effects of KA on MHCC97H and SMMC 7721 cells, we first measured the IC_50_ of KA in these cell lines using the MTT method after treating the tumor cells with a series of gradient concentrations of KA for 48 h (Figure 2A). After statistical calculation, the IC_50_ value of KA in MHCC97 H was determined to be 8.40 µM, and in SMMC 7721 was 9.85 µM. Based on these IC_50_ values, we set the concentration gradients for subsequent experiments. Continuous MTT assays over 7 days showed that KA significantly inhibited HCC cell proliferation in a dose-dependent manner compared to the DMSO control group (Figure 2B). Colony formation assays also showed significantly reduced colony formation ability in KA-treated groups compared to controls. Even at a relatively low concentration of KA, the number of colonies had significantly reduced. (Figure 2C). Similarly, EdU assays revealed a significant decrease in the proportion of proliferating HCC cells after 48 h of KA treatment (Figure 2D). These results confirmed the antiproliferative effect of KA in vitro. In addition, the MHCC97 H cells labeled with luciferase were injected through the tail vein to explore the pulmonary metastasis of HCC cells by the in vivo imaging method. HCC cells enter the heart via the iliac vein and inferior vena cava through the bloodstream. Subsequently, these highly metastatic HCC cells are transported through the pulmonary artery and become enriched in the lungs. Three days after the injection of HCC cells, intraperitoneal injection of KA (30 mg/kg) or DMSO was performed. Administered the drug once every two days for a total of 32 days. The results indicated that KA treatment significantly inhibited the pulmonary metastasis (Figure 2E). To further elucidate the in vivo effects of KA, we injected the MHCC97H cells into the livers of BALB/c-nu mice and monitored the survival status. The administration of KA was as described previously, and the administration period was extended to 45 days. It showed that the survival time in KA-treated animals had been significantly extended (Figure 2F). Additionally, renal and liver function tests showed no significant changes in KA-treated mice compared to controls (Figure 2G). These experimental results demonstrated that KA effectively inhibited HCC cell proliferation both in vitro and in vivo and had a notable impact on liver cancer pulmonary metastasis in animal models.

### 3.3. KA Induced Cell Cycle Arrest and Migration/Invasion Inhibition in HCC Cells

**Figure 3:**
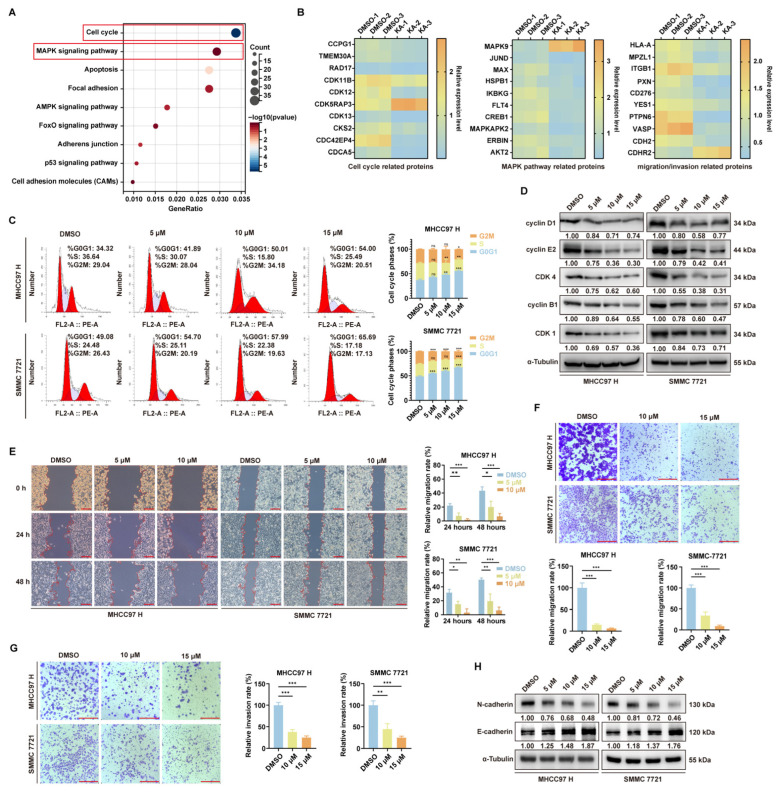
Kuwanon A induced the cycle arrest and migration/invasion inhibition. (**A**) KEGG analysis of genes down or upregulated (determined by proteomics data) in MHCC97 H after it was treated with KA (10 μM). The cell cycle and MAPK pathway were marked by a red box. The DMSO groups were used as controls. (**B**) According to the proteomics data, the regulation of some proteins related to the main pathway is detailed. The DMSO groups were used as controls. (**C**) The flow cytometry assays were used to detect the cell cycle progress of HCC cells (MHCC97 H and SMMC 7721) after treatment with the indicated concentration of KA for 48 h. The DMSO groups were used as controls. (**D**) Western blot assays were used to determine the level of CDK and cyclin proteins in HCC cells (MHCC97 H and SMMC 7721), which were treated with the indicated concentration of KA for 48 h. The DMSO groups were used as controls. (**E**) The wound healing assay was used to detect the migration ability of highly metastatic HCC cells (MHCC97 H and SMMC 7721) after treatment with the indicated concentration of KA for 48 h. The DMSO groups were used as controls. Scale bars = 200 μm (**F**). The Transwell without Matrix-Gel and (**G**) with Matrix-Gel were used to detect the migration and invasion ability of highly metastatic HCC cells (MHCC97 H and SMMC 7721) after treatment with the indicated concentration of KA for 48 h. The DMSO groups were used as controls. Scale bars = 200 μm (**H**). Western blot assays were used to determine the level of N-cadherin and E-cadherin in HCC cells (MHCC97 H and SMMC 7721), which were treated with the indicated concentration of KA for 48 h. The DMSO groups were used as controls. All data are shown as the means ± SD; * *p* < 0.05, ** *p* < 0.01, *** *p* < 0.001. ns: not significant.

To elucidate the mechanism by which KA affects the proliferation and migration of HCC cells, we compared and enriched the proteins with altered expression levels in proteomic analyses after MHCC97 H cells were treated with KA. Revealing significant changes in proteins related to cell cycle, MAPK signaling pathway, apoptosis, focal adhesion, and so on (Figure 3A). Considering the previous results and proteomics data, we focused on the cell cycle progression and migration/invasion. These biological process-related proteins in the proteomic data were quantitatively analyzed in the form of heat maps (Figure 3B). Flow cytometry analysis of the cell cycle showed significant G0/G1 phase arrest in HCC cells treated with KA in a concentration-dependent manner (Figure 3C). Western blot results confirmed the downregulation of corresponding cell cycle-related proteins, indicating that KA affects cell cycle progression (Figure 3D). Subsequently, cell scratch assays demonstrated a significant reduction in wound healing in KA-treated groups compared to controls (Figure 3E). The Transwell assays also showed a significant decrease in the number of HCC cells migrating through the polycarbonate membrane after KA treatment (Figure 3F). Additionally, KA also inhibited the invasive capacity of HCC cells when Matrix-Gel was added to the Transwell chambers (Figure 3G). Western blot analysis of epithelial-mesenchymal transition (EMT) markers revealed downregulation of N-cadherin and upregulation of E-cadherin (Figure 3H). These results indicated that KA induced the G0/G1 cell cycle arrest and inhibited EMT in HCC cells.

### 3.4. KA Directly Targeted to YWHAB and Induced the Dissociation of Protein Dimerization

**Figure 4:**
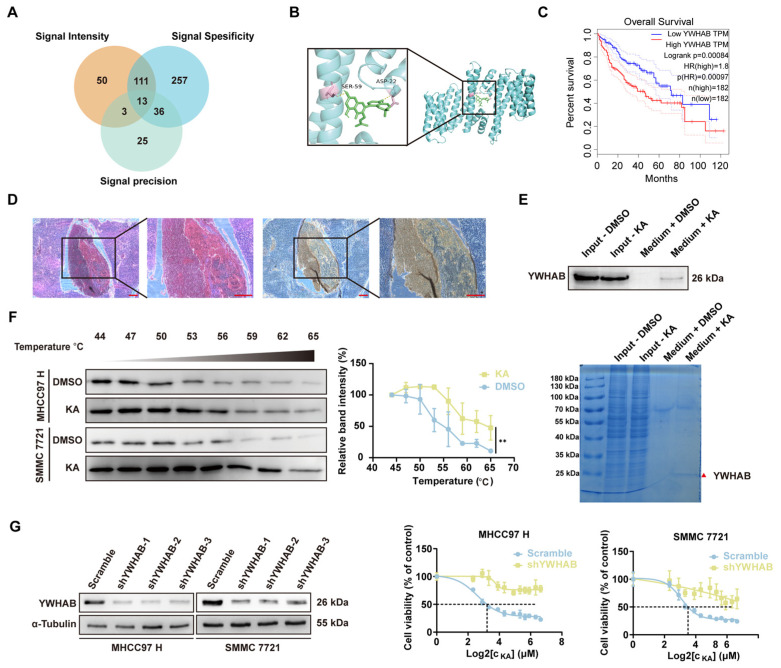
Kuwanon A directly targeted YWHAB. (**A**) Identified the specific target protein of KA through the LC-MS/MS data. The relevant data on signal intensity, specificity, and precision were analyzed separately. Then, the intersection was obtained through the Venn diagram. A list of 13 interacting proteins was presented in the supplementary Table S3. (**B**) The interaction location between KA and target protein YWHAB was predicted by AutoDock, and PyMOL was used for visual analysis. The protein in blue indicated YWHAB, and the molecule in green was KA. The definite amino acids that interact with KA are in pink (SER-59 and ASP-22). (**C**) The GEPIA database indicated the high expression of YHWAB related to the poor prognosis of HCC. (**D**) IHC and H&E stain were used to detect YWHAB expression level in orthotopic transplanted tumor of mice and para-cancerous tissue. Scale bars = 200 μm (**E**). The Sepharose 6B pull-down assay was performed in HEK 293T, which overexpressed YWHAB. Results showed both by Western Blot assay (above) and Coomassie brilliant blue staining (below). The red triangles in the Coomassie brilliant blue staining figure indicated the target protein YWHAB. The medium + DMSO group was used as a control. (**F**) The CETSA assay was performed in HCC cells (MHCC97 H and SMMC 7721) after treatment with a high concentration of KA for 3 h. The detection of protein thermal stability was used to reflect the binding of the compound to the protein. The DMSO groups were used as controls. (**G**) The Western blot (left part) was used to determine the interference efficiency of YWHAB in knockdown cell lines, and the most effective one was chosen to detect the IC50 of KA (right part). The gradient concentration of KA (0, 5, 7.5, 10, 15, 20, 30, 40, 50, 60, 80, 100 μM) treated cells for 48 h, followed by the MTT method to detect the cell viability. The scramble groups were used as control. All data are shown as the means ± SD; ** *p* < 0.01.

**Figure 5:**
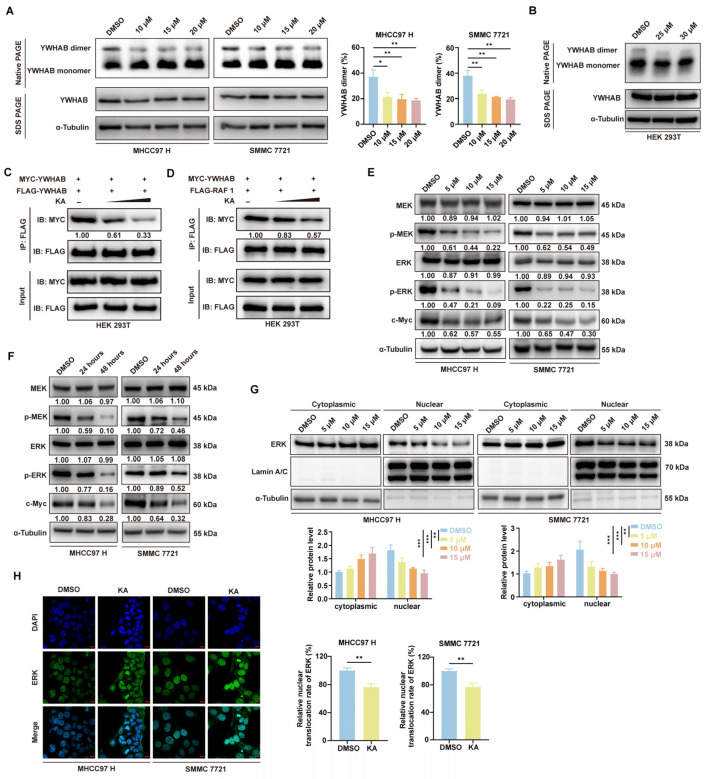
Kuwanon A repressed the MAPK pathway by inducing the depolymerization of YWHAB. (**A**) The Blue-Native PAGE Running assay was used to detect the level of YWHAB dimer in HCC cells (MHCC97 H and SMMC 7721) after treatment with the indicated concentration of KA. The expression level of YWHAB and α-Tubulin was detected through SDS-PAGE Running assay. The DMSO groups were used as controls. (**B**) The Blue-Native PAGE Running assay was used to detect the level of YWHAB dimer in HEK 293T cells, which overexpressed FLAG-YWHAB, after treatment with a gradient concentration of KA. The DMSO group was used as a control. (**C**) The 25 μM and 30 μM of KA were used to treat the HEK 293T cells, which overexpressed MYC-YWAHB and FLAG-YWHAB. The FLAG-YWHAB was pulled down by anti-FLAG antibody and immunoblotted with anti-MYC antibody. The DMSO group was used as a control. (**D**) The 25 μM and 30 μM of KA were used to treat the HEK 293T cells, which overexpressed MYC-YWHAB and FLAG-Raf 1. The FLAG-Raf 1 was pulled down by anti-FLAG antibody and immunoblotted with anti-MYC antibody. The DMSO group was used as a control. (**E**) Western blot assays were used to determine the level of MEK, pMEK, ERK, pERK, and c-Myc in HCC cells (MHCC97 H and SMMC 7721), which were treated with the indicated concentration of KA for 48 h. The DMSO groups were used as controls. (**F**) Western blot assays were used to determine the level of MEK, pMEK, ERK, pERK, and c-Myc in HCC cells (MHCC97 H and SMMC 7721), which were treated with KA (15 μM) for 24 or 48 h. The DMSO groups were used as controls. (**G**) The amount of ERK in the cytoplasm and the nucleus was analyzed, respectively, through the Nuclear and Cytoplasmic Protein Extraction assay after HCC cells were treated with the indicated concentration of KA for 48 h. The DMSO groups were used as controls. (**H**) The localization of ERK was investigated by immunofluorescence staining and observed by confocal microscope after HCC cells were treated with KA (10 μM) or DMSO for 48 h. The DMSO groups were used as controls. Scale bars = 10 μm. All data are shown as the means ± SD; * *p* < 0.05, ** *p* < 0.01, *** *p* < 0.001.

To identify the direct target of KA in HCC cells, we immobilized KA on epoxide-activated Sepharose 6B media (Cytia, Washington, DC, USA) and used mass spectrometry to analyze the proteins that directly interacted with the small molecule drug. Among the identified proteins, we focused on YWHAB, a member of the 14-3-3 protein family (Figure 4A, Supplementary Table S3). Subsequently, we predicted the drug pocket of YWHAB and conducted AutoDock molecular docking as well as PyMOL visualization analysis (Figure 4B). The result suggested that KA probably occupied the dimerization binding site of YWHAB. Next, we explored the clinical significance of this protein through the GEPIA database (http://gepia.cancer-pku.cn/) (accessed on 18 May 2024). It indicated a significant correlation between high expression of this protein and poor prognosis in patients (Figure 4C). The immunohistochemistry also showed higher expression of YWHAB in the mouse xenograft tumor compared to adjacent tissue (Figure 4D). The pulldown experiments confirmed the direct interaction between KA and YWHAB (Figure 4E). Cellular thermal shift assays demonstrated increased thermal stability of YWHAB in KA-treated HCC cells compared to controls (Figure 4F). It further proved the interaction between KA and YWHAB. Subsequent knockdown of YWHAB in HCC cells revealed a significant increase in the IC_50_ of KA (Figure 4G, Supplementary Table S1). It confirmed that YWHAB played a crucial role in enabling KA to perform its biological functions. Because of the widely accepted conclusion that the protein function of YWHAB relies on the dimeric form, we investigated the effect of KA on the dimerization of YWHAB. Native-PAGE electrophoresis showed that the proportion of YWHAB dimer had decreased in the treated groups. This result is consistent in both HCC cell lines and FLAG-YWHAB overexpressed HEK 293T cells (Figure 5A,B). These results validated the AutoDock prediction that KA disrupted the dimerization of YWHAB. Co-immunoprecipitation experiments in HEK 293T cells overexpressing YWHAB with different tags also indicated reduced dimerization levels of YWHAB after KA treatment (Figure 5C). It further proves that KA directly targets YWHAB and induces its depolymerization.

### 3.5. KA Blocked the Phosphorylation Signal Transmission of the Raf/MEK/ERK Pathway

Previous studies have implied the connection between YWHAB and the MAPK signaling pathway [22]. Researchers proposed that the YWHAB dimer is necessary to support Raf1 activity, even though Raf1 contained two independent and high-affinity binding sites [23]. Based on proteomic analysis and existing research, we hypothesized that the YWHAB dissociation caused by KA will further affect the activity of Raf1. So, we investigated the effects of KA on the interaction between Raf1 and YWHAB. Co-immunoprecipitation results showed a reduction in the amount of YWHAB interacting with Raf1 (Figure 5D). Subsequent Western blot analysis revealed significant downregulation of the phosphorylation levels of MEK1/2 and ERK1/2 (Figure 5E,F). These results demonstrated that KA affected the activity of Raf1 and the MAPK signaling pathway. Next, we investigated the nuclear translocation of ERK. Nuclear and cytoplasmic protein extraction assays indicated that ERK accumulated in the cytoplasm and reduced nuclear translocation after KA treatment (Figure 5G). To further confirm the subcellular localization of ERK, immunofluorescence staining was performed, and confocal microscopy observations showed that KA inhibited the nuclear translocation of ERK (Figure 5H). These results demonstrated that KA blocked the phosphorylation signal transmission of the Raf/MEK/ERK pathway by disturbing YWHAB dimer formation.

### 3.6. Overexpression of YWHAB Partly Reversed KA-Induced Cell Cycle Arrest, Proliferation, and Migration/Invasion Inhibition

**Figure 6:**
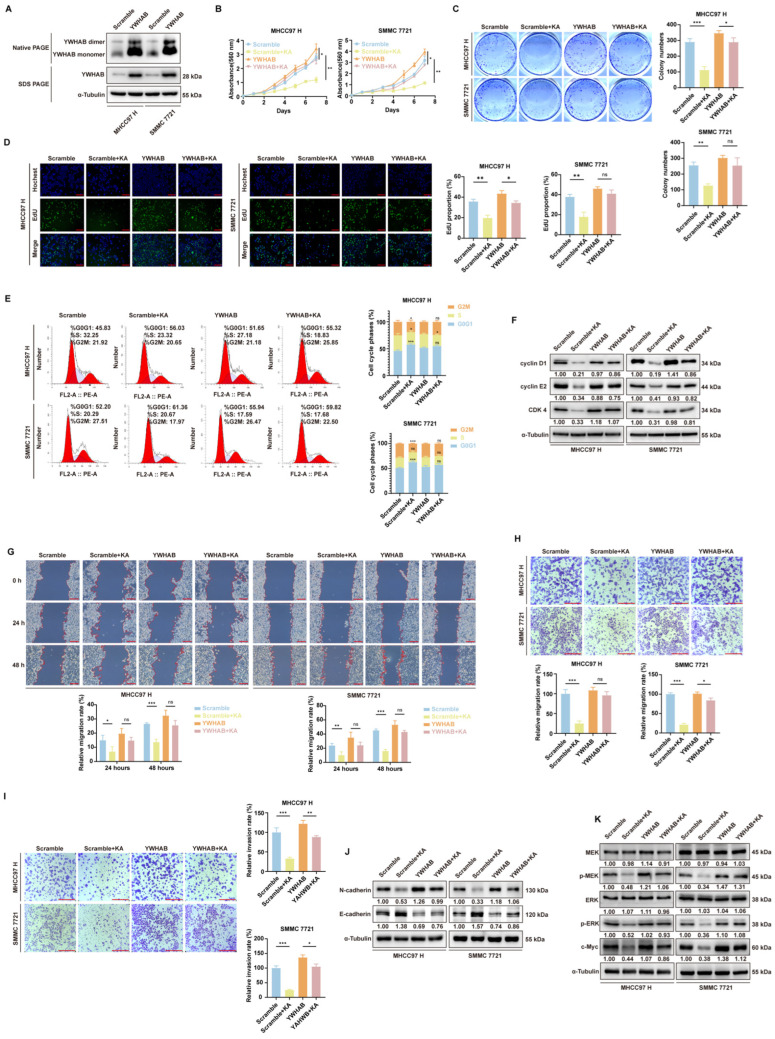
The functions of Kuwanon A were partly reversed by YWHAB overexpression. (**A**) The Western Blot and Blue-Native PAGE Running assay were used to determine the level of YWHAB in overexpression HCC cell lines. The scramble groups were used as controls. (**B**) The MTT method was used to detect cell viability in YWHAB overexpression cell lines (MHCC97 H and SMMC 7721) after treatment with KA (15 μM) or DMSO. The absorption at 560 nm was detected after treating 1, 2, 3, 4, 5, 6, and 7 days. The scramble groups were used as controls. (**C**) In overexpression cell lines (MHCC97 H and SMMC 7721), the ability of colony formation in vitro was determined after KA (15 μM) or DMSO treatment. The scramble groups were used as controls. (**D**) The EdU assay was used to determine the amplification ability of YWHAB overexpression cell lines (MHCC97 H and SMMC 7721) after treatment with KA (15 μM) or DMSO for 48 h. The vector groups were used as controls. Scale bars = 200 μm (**E**). The flow cytometry assays were used to determine the cell cycle progression in YWHAB overexpression cell lines (MHCC97 H and SMMC 7721) after treatment with KA (15 μM) or DMSO for 48 h. The scramble groups were used as controls. (**F**) Western blot assays were used to determine the expression level of cyclin D1, cyclin E2, and CDK 4 in overexpression cell lines (MHCC97 H and SMMC 7721), which were treated with KA (15 μM) or DMSO for 48 h. The scramble groups were used as controls. (**G**) The wound healing assay was used to detect the migration ability of overexpression cell lines (MHCC97 H and SMMC 7721) after treatment with 15 μM KA or DMSO for 48 h. The scramble groups were used as control. Scale bars = 200 μm (**H**). The Transwell without Matrix-Gel and (**I**) with Matrix-Gel were used to detect the migration and invasion ability of overexpression cell lines (MHCC97 H and SMMC 7721) after treatment with KA (15 μM) or DMSO for 48 h. The scramble groups were used as control. Scale bars = 200 μm (**J**) Western blot assays were used to determine the level of N-cadherin and E-cadherin in overexpression cell lines (MHCC97 H and SMMC 7721), which were treated with KA (15 μM) or DMSO for 48 h. The scramble groups were used as controls. (**K**) Western blot assays were used to determine the level of MEK, pMEK, ERK, pERK, and c-Myc in overexpression cell lines (MHCC97 H and SMMC 7721), which were treated with KA (15 μM) or DMSO for 48 h. The scramble groups were used as controls. All data are shown as the means ± SD; * *p* < 0.05, ** *p* < 0.01, *** *p* < 0.001. ns: not significant.

In this part, we explored the significance of YWHAB in enabling the function of KA. According to the existing results, KA inhibited the dimerization of YWHAB. Therefore, in this section, we increased the level of the dimer by overexpressing YWHAB. We established stable YWHAB-overexpressing HCC cell lines using lentiviral infection (Figure 6A). MTT and EdU assays showed that YWHAB overexpression partly reversed the proliferation inhibition caused by KA in HCC cells (Figure 6B,D). Colony formation assays also indicated that KA failed to inhibit the in vitro colony formation ability of YWHAB-overexpressing HCC cells (Figure 6C). It showed that YWHAB was involved in KA-mediated proliferation inhibition. Flow cytometry analysis of cell cycle progression revealed less KA-induced G0/G1 phase arrest in YWHAB-overexpressing cells (Figure 6E). Western blot analysis of G0/G1 phase cell cycle proteins showed similar results (Figure 6F). It further proved the significant importance of YWHAB for the biological functions of KA. Cell wound healing and Transwell assays demonstrated less effect of KA on the migration/invasion of YWHAB-overexpressing HCC cells compared to normal tumor cells (Figure 6G–I). Western blot analysis revealed that KA treatment induced minimal changes in the N-cadherin/E-cadherin ratio in YWHAB-overexpressing HCC cells. (Figure 6J). These results indicated that YWHAB was crucial for KA-induced migration inhibition. Additionally, analysis of the MAPK pathway by Western Blot demonstrated that YWHAB overexpression partly abrogated KA’s effects on MEK1/2 and ERK1/2 phosphorylation (Figure 6K). These results further demonstrated that KA exerted its effects on HCC cell proliferation, cycle progression, and migration/invasion by interfering with YWHAB protein function.

### 3.7. Synergistic Effects of KA and Sorafenib on the Inhibition of Cell Proliferation and Migration/Invasion

**Figure 7:**
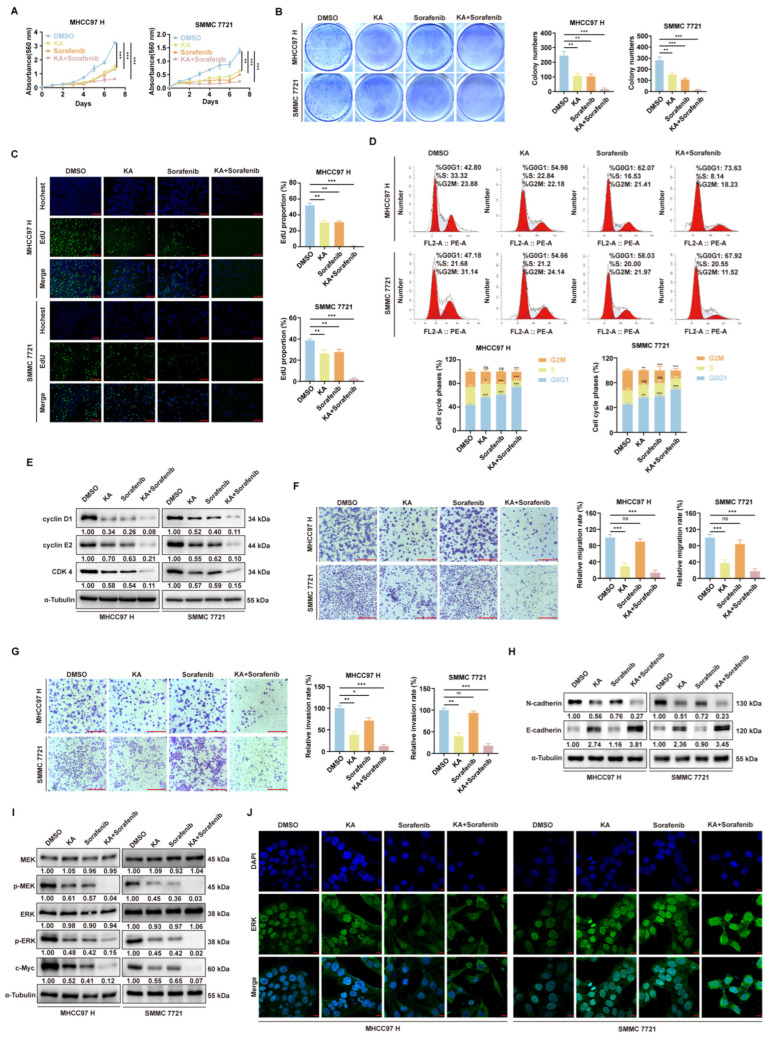
The combination of Kuwanon A and Sorafenib has a synergistic effect in the proliferation, migration, and invasion inhibition of HCC cells. (**A**) The MTT assay was used to detect the cell viability of HCC cells (MHCC97 H and SMMC 7721), which had been treated with KA (10 μM), Sorafenib (10 μM), or combined medication (10 μM KA and 10 μM Sorafenib) for 2 days. The DMSO groups were used as controls. (**B**) In HCC cells (MHCC97 H and SMMC 7721), the effect of KA (10 μM), Sorafenib (10 μM), or combined medication (10 μM KA and 10 μM Sorafenib) on colony formation in vitro was determined. The DMSO groups were used as controls. (**C**) The EdU assay was used to determine the amplification ability of HCC cells (MHCC97 H and SMMC 7721) after treatment with KA (10 μM), Sorafenib (10 μM), or combined medication (10 μM KA and 10 μM Sorafenib) for 48 h. The DMSO groups were used as controls. Scale bars = 200 μm (**D**). The flow cytometry assays were used to determine the cell cycle progression of HCC cells (MHCC97 H and SMMC 7721) after treatment with KA (10 μM), Sorafenib (10 μM), or combined medication (10 μM KA and 10 μM Sorafenib) for 48 h. The DMSO groups were used as controls. (**E**) Western blot assays were used to determine the expression level of cyclin D1, cyclin E2, and CDK 4 in HCC cells (MHCC97 H and SMMC 7721) after treatment with KA (10 μM), Sorafenib (10 μM), or combination treatment (10 μM KA and 10 μM Sorafenib) for 48 h. The DMSO groups were used as controls. (**F**) The Transwell without Matrix-Gel and (**G**) with Matrix-Gel were used to detect the migration and invasion ability of HCC cells (MHCC97 H and SMMC 7721) after treatment with KA (10 μM), Sorafenib (10 μM), or combined medication (10 μM KA and 10 μM Sorafenib) for 48 h. The DMSO groups were used as controls. Scale bars = 200 μm (**H**) Western blot assays were used to determine the level of N-cadherin and E-cadherin in HCC cells (MHCC97 H and SMMC 7721) after treatment with KA (10 μM), Sorafenib (10 μM), or combined medication (10 μM KA and 10 μM Sorafenib) for 48 h. The DMSO groups were used as controls. (**I**) Western blot assays were used to determine the level of MEK, pMEK, ERK, pERK, and c-Myc in HCC cells (MHCC97 H and SMMC 7721) after treatment with KA (10 μM), Sorafenib (10 μM), or combined medication (10 μM KA and 10 μM Sorafenib) for 48 h. The DMSO groups were used as controls. (**J**) The localization of ERK was investigated by immunofluorescence staining and observed by confocal microscope after HCC cells were treated with KA (10 μM), Sorafenib (10 μM), or combined medication (10 μM KA and 10 μM Sorafenib) for 48 h. The DMSO groups were used as controls. Scale bars = 10 μm All data are shown as the means ± SD; * *p* < 0.05, ** *p* < 0.01, *** *p* < 0.001. ns: not significant.

Sorafenib is a clinical drug used for HCC treatment. It has been clearly identified that it is an inhibitor of the MAPK signaling pathway [24]. Considering that KA had a similar anti-tumor mechanism, we explored the combined effects of KA and Sorafenib on HCC cells. Refer to the data provided on the MCE website (https://www.medchemexpress.cn/) (accessed on 15 February 2025), the IC_50_ of Sorafenib in both MHCC97 H and SMMC 7721 were around 10 µM. Therefore, in the subsequent experiments, the working concentration of Sorafenib was designated as 10 µM. Firstly, the MTT and EdU assays showed that the combination treatment significantly inhibited cell proliferation with a synergistic effect (Figure 7A,C). Colony formation assays also revealed a significant reduction in the number of in vitro colonies formed by HCC cells after combination treatment (Figure 7B). It demonstrated the good effect of combination treatment in the inhibition of HCC cell proliferation. Flow cytometry and Western blot analyses showed that the combination treatment induced more significant G0/G1 phase cell cycle arrest compared to KA or sorafenib alone (Figure 7D,E). Transwell assays indicated that a low concentration of sorafenib alone had no significant effect on HCC cell migration/invasion, whereas the combination treatment significantly reduced the number of cells migrating through the polycarbonate membrane or Matrigel (Figure 7F,G). These results indicated that KA enhanced the migration-inhibitory effects of sorafenib. Analysis of N-cadherin and E-cadherin expression levels showed similar results (Figure 7H). Therefore, it proved that KA and Sorafenib also had an excellent synergistic effect in the inhibition of migration and invasion. Additionally, Western blot analysis revealed significant inhibition of MEK1/2 and ERK1/2 phosphorylation levels after combination treatment (Figure 7I). Immunofluorescence signal analysis of ERK protein localization also showed that the nuclear translocation of ERK was more significantly affected by the combination treatment (Figure 7J, Supplementary Figure S2). It showed that the combination treatment also had a well-synergistic effect in blocking the MAPK signaling pathway. In conclusion, the above results demonstrated the synergistic effect of combination treatment in MAPK pathway blocking, as well as the inhibition of proliferation, migration, and invasion in HCC cells.

## 4. Discussion

In our research, we provided mechanistic insights into the anticancer effects of Kuwanon A in hepatocellular carcinoma cells. Through compound bank screening, we selected KA and further characterized YWHAB as its target protein using LC-MS/MS. We demonstrated that KA repressed the MAPK pathway, which had been enriched in proteomics, by directly targeting YWHAB and disrupting its dimerization. It suggested that KA effectively blocked the Raf/MEK/ERK signaling cascade, which was crucial for cell cycle progression and migration/invasion of HCC cells. Additionally, the combination of KA with sorafenib, a clinically approved drug for HCC treatment, demonstrated significant synergistic effects on the inhibition of cell proliferation, migration, and invasion. The enhanced inhibition of MEK1/2 and ERK1/2 phosphorylation observed in the combination treatment suggests that KA may potentiate the effects of sorafenib by targeting overlapping pathways [25,26]. In a word, our research has supported the anti-proliferative and anti-metastatic capabilities of KA in HCC.

Hepatocellular carcinoma (HCC) accounts for up to 90% of all liver cancer cases, with the vast majority developing from pre-existing chronic liver diseases. In developing countries, hepatitis B virus (HBV) and hepatitis C virus (HCV) infections are major contributors to HCC, whereas in developed countries, non-alcoholic steatohepatitis (NASH) plays a more prominent role [4,5]. Despite recent advances in diagnostic techniques and therapeutic strategies, the prognosis for HCC patients remains poor, largely due to the high frequency of metastasis and recurrence [27]. The most common sites of HCC metastasis include the lungs, lymph nodes, and bones, with pulmonary metastasis being particularly frequent [27,28]. This metastatic potential significantly contributes to the high mortality rate associated with HCC.

The current treatment options for HCC are limited and often insufficient to achieve long-term remission. Surgical resection and liver transplantation are the primary curative treatments, but they are only suitable for a minority of patients with early-stage disease. For advanced HCC, targeted therapies such as sorafenib and lenvatinib have shown some efficacy, but their benefits are often modest, and resistance frequently develops [29,30]. Immunotherapies, including immune checkpoint inhibitors, have also shown promise, but their response rates are variable, and they are associated with significant side effects [31]. Therefore, there is an urgent need for novel therapeutic agents that can effectively inhibit HCC progression and metastasis, particularly in high-risk populations. Our research also focused on this aspect and investigated the role of the mulberry active substances in enhancing the sensitivity to chemotherapy. We have revealed that the combination of KA with sorafenib can significantly inhibit the proliferation and epithelial-mesenchymal transition (EMT) of HCC cells, as evidenced by a series of experiments. In our research, we found that the combination of KA with sorafenib can significantly exhibit anti-migration and anti-invasion capabilities that sorafenib alone did not possess. Previous studies on the functions of sorafenib have mainly focused on its role in angiogenesis and proliferation inhibition. An important study on the mechanism of sorafenib’s action had suggested that sorafenib is a multi-kinase inhibitor, including Raf kinase and various receptor tyrosine kinases [24]. Numerous early studies have demonstrated that sorafenib inhibited the proliferation of HCC cells by blocking the Raf/MEK/ERK signaling pathway and decreased microvascular density by suppressing VEGFR and PDGFR signaling [32,33,34,35]. However, there has been little discussion about the role of sorafenib in inhibiting migration and invasion or in preventing pulmonary metastasis. At the same time, recent studies on sorafenib resistance have proposed various mechanisms for the migration and invasion of resistant HCC cells. There are some studies focused on the inhibition of the mTOR-related signaling pathway [36]. At the same time, some studies had also suggested that inhibiting the accumulation of c-Myc is associated with overcoming sorafenib resistance in advanced HCC patients [37], which was in line with our research findings. KA can enhance the inhibitory effect of sorafenib on c-Myc expression, as well as the migration and invasion of HCC cells, which further supports its potential as an anti-metastatic agent. However, this method does not explain why sorafenib, when administered as a monotherapy, can effectively suppress the expression of c-Myc but has no significant impact on the levels of N-cadherin and E-cadherin. This suggested that sorafenib, as a multi-kinase inhibitor, may modulate EMT-related proteins and migration through alternative mechanisms, such as the activation of the STAT3 or EGFR/beta-catenin pathway, as mentioned in some studies [38,39]. Further investigation into the broader mechanisms of sorafenib may contribute to overcoming chemotherapeutic resistance in HCC.

The research on Kuwanon A in tumors is not extensive, though there are articles suggesting its anti-tumor function in melanoma and gastric cancer [9,10]. KA might suppress melanoma cell proliferation and migration by promoting β-catenin protein degradation via the E3 ubiquitin ligase synoviolin 1 (SYVN1) [9]. However, there was no direct evidence to prove that SYVN1 was the specific target of KA. The other research revealed that KA could induce unfolded protein response (UPR) and endoplasmic reticulum (ER) stress, potentially resulting in DNA damage, autophagy, and cell death [10]. But no detailed discussion was conducted regarding the mechanism. Our research not only supplemented the functional study of KA in HCC but also analyzed the protein targets and mechanisms. We have demonstrated the interaction between YWHAB and KA in detail. It also fully elaborated that KA inhibited the proliferation, migration, and invasion of HCC cells by suppressing the MAPK signaling pathway.

The target protein YWHAB mentioned in our research has been involved in many studies, including investigations related to neurodegenerative diseases and some kinds of cancer. A study on Alzheimer’s disease found significant associations between cerebrospinal fluid (CSF) 14-3-3β levels and CSF biomarkers of p-tau, t-tau, pTau/Aβ42 ratios, and GAP-43, as well as other Alzheimer’s disease biomarkers such as Aβ-PET. Authors proposed the association between CSF 14-3-3β and progressive cognitive decline in Alzheimer’s disease [40]. In breast cancer cells, IRX5 could inhibit the migration and invasion of cells by inducing YWHAB, which contradicts our research findings [13]. However, more research has shown that high levels of YWHAB promote tumor progression. A study related to colon cancer had proved that YWHAB knockdown inhibited cell proliferation whilst promoting cell cycle arrest and apoptosis through PIK3R2 [14]. Additionally, the reduction of YWHAB contributed to etoposide-induced lung cancer apoptosis [17]. What is more, the identification and validation of novel biomarkers of HCC had demonstrated that YWHAB was upregulated in HCC samples, and higher expression levels were associated with advanced tumor stages and T grades [15]. Regarding this discrepancy, we have conducted some analyses and suppositions. As YWHAB has a strong affinity for proteins containing phosphorylated serine or phosphorylated threonine, it is involved in regulating numerous signaling pathways and protein function [12]. As a result, depending on the specific biological context, YWHAB can exhibit dual functional roles, either promoting or suppressing tumorigenesis. For instance, YWHAB regulated the Hippo pathway by interacting and promoting the cytoplasmic retention of YAP [41]. There is also evidence suggesting that YWHAB can activate the PI3K/AKT pathway and promote tumor progression. These findings suggested that YWHAB may have pleiotropic functions, potentially exerting different effects under varying conditions or cellular contexts.

Our research still has some limitations. To make the conclusions more rigorous, animal experiments used to prove the synergistic effect of KA and sorafenib in inhibiting the proliferation and migration/invasion of HCC in vivo are necessary. Additionally, more research is needed to explore how KA can address sorafenib’s limitation in migration inhibition during combined medication. Additionally, this study was conducted solely in cell cultures and mice models. Our findings indicated the potential and promise of Kuwanon A, a bioactive compound from mulberry, as an anticancer agent. However, its clinical relevance remains to be substantiated by more extensive data and further research.

In conclusion, our study demonstrated that KA, a natural compound derived from mulberry root bark, had significant anticancer effects in HCC cells by targeting YWHAB and disrupting the MAPK signaling pathway. The synergistic interaction with sorafenib further highlighted its potential as a therapeutic agent. These findings provided a possibility for further investigation of KA as a novel therapeutic option for HCC.

## Data Availability

The datasets used and/or analyzed during the current study are available from the corresponding author on reasonable request.

## Supplementary Materials

The following supporting information can be downloaded at https://www.mdpi.com/article/10.3390/cells14191487/s1: Figure S1: The number of the mulberry active substances, which were represented as the orange dots in Figure 1C; Figure S2: Quantitative analysis of the effect of combined medication on ERK nuclear translocation (Figure 7J). All data are shown as the means ± SD; * *p* < 0.05, ** *p* < 0.01, *** *p* < 0.001; Table S1: The shRNA sequences were listed as below; Table S2: Table of Correspondence between the NO. and Name of the Mulberry Active Substances; Table S3: The Proteins interacting with KA according to the LC-MS/MS data (Sort in order of priority).

## Abbreviations

The following abbreviations are used in this manuscript:

HCC	Hepatocellular carcinoma
KA	Kuwanon A
YWHAB	Tyrosine 3-monooxygenase/tryptophan 5-monooxygenase activation protein beta
MTT	Methylthiazolyldiphenyl-tetrazolium bromide
CCK8	Cell Counting Kit-8
EdU	5-Ethynyl-2′-deoxyuridine
WB	Western Blot
CETSA	Cell thermal assay
BN-PAGE	Blue native polyacrylamide gel electrophoresis
H&E	Hematoxylin-Eosin staining
IHC	Immunohistochemistry
MAPK	Mitogen-activated protein kinase
MEK	Mitogen-activated extracellular signal-regulated kinase
ERK	Extracellular regulated protein kinases
