# Supplementary material for: Kuwanon A Targeted YWHAB in Hepatocellular Carcinoma Cells to Inhibit the Raf/MEK/ERK Signaling Pathway
Source: Cells. 2025 Sep 23;14(19):1487. doi: 10.3390/cells14191487 (PMC12523843; doi:10.3390/cells14191487)
Supplement: Supplementary file 1 [file cells-14-01487-s001.zip › supplementary Table S1.pdf]

Supplementary Table S1. The shRNA sequences were listed as below:

shYWHAB-1-F	CCGGCGCTGAATGAAGAGTCTTATACTCGAGTATAAGACTCTTCATTCAGCGTTTTTG
shYWHAB-1-R	AATTCAAAAACGCTGAATGAAGAGTCTTATACTCGAGTATAAGACTCTTCATTCAGCG
shYWHAB-2-F	CCGGGCTGAATTGGATACGCTGAATCTCGAGATTCAGCGTATCCAATTCAGCTTTTTG
shYWHAB-2-R	AATTCAAAAAGCTGAATTGGATACGCTGAATCTCGAGATTCAGCGTATCCAATTCAGC
shYWHAB-3-F	CCGGCCCAATGCTACACAACCAGAACTCGAGTTCTGGTTGTGTAGCATTGGGTTTTTG
shYWHAB-3-R	AATTCAAAAACCCAATGCTACACAACCAGAACTCGAGTTCTGGTTGTGTAGCATTGGG
