# Supplementary figures and images for: Kuwanon A Targeted YWHAB in Hepatocellular Carcinoma Cells to Inhibit the Raf/MEK/ERK Signaling Pathway
Source: Cells. 2025 Sep 23;14(19):1487. doi: 10.3390/cells14191487 (PMC12523843; doi:10.3390/cells14191487)

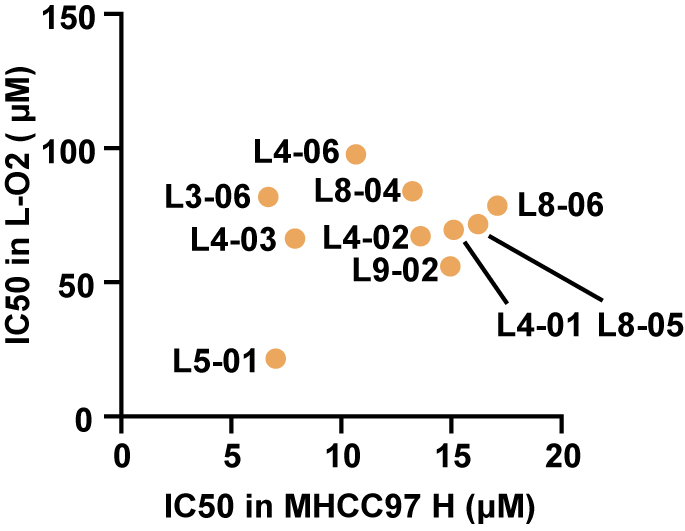

Supplement: Supplementary file 1 [file cells-14-01487-s001.zip › supplementary Figure S1.tif]

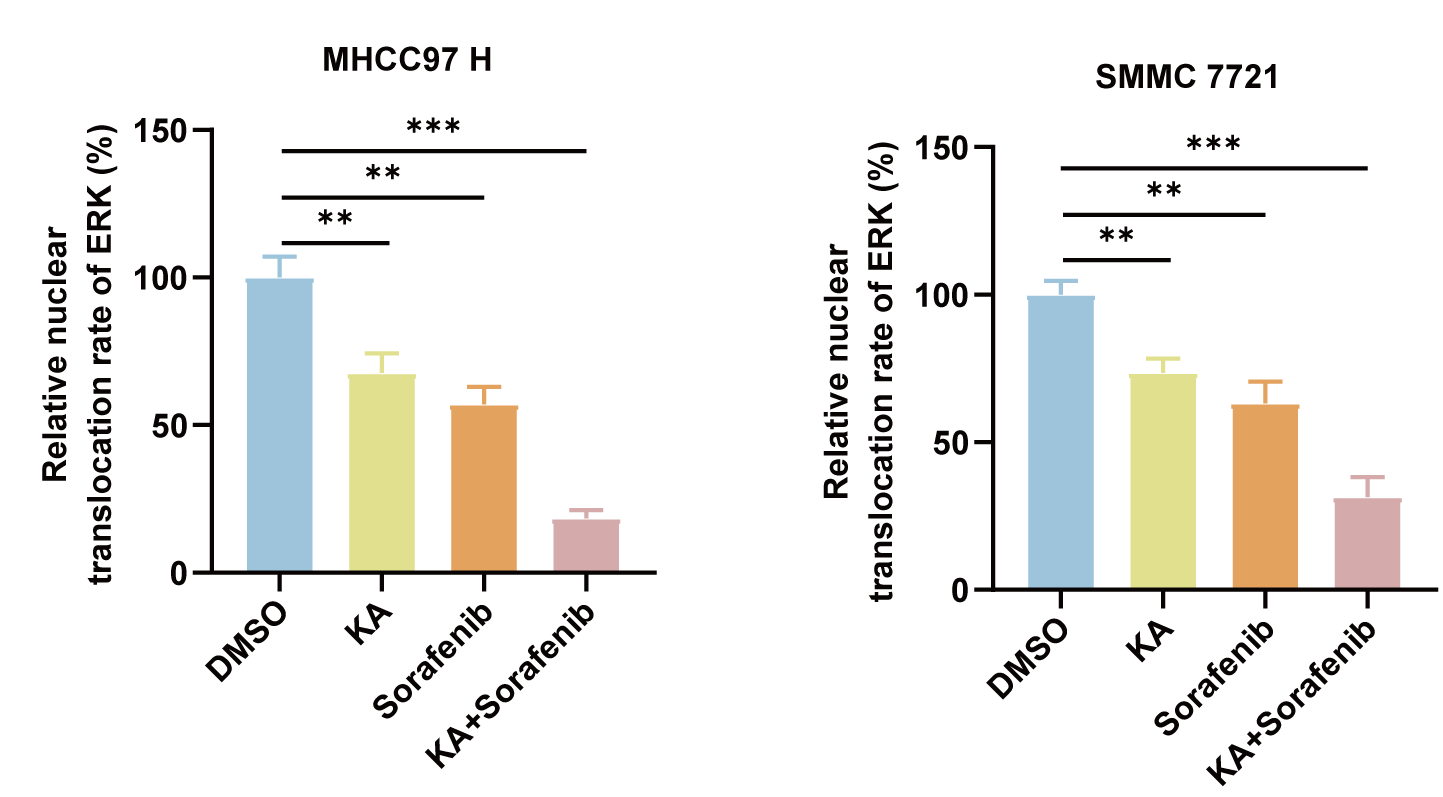

Supplement: Supplementary file 1 [file cells-14-01487-s001.zip › supplementary Figure S2.tif]
